# Combinational Immunotherapy with Allo-DRibble Vaccines and Anti-OX40 Co-Stimulation Leads to Generation of Cross-Reactive Effector T Cells and Tumor Regression

**DOI:** 10.1038/srep37558

**Published:** 2016-11-22

**Authors:** Guangjie Yu, Yuhuan Li, Zhihua Cui, Nicholas P. Morris, Andrew D. Weinberg, Bernard A. Fox, Walter J. Urba, Lixin Wang, Hong-Ming Hu

**Affiliations:** 1 School of Medicine, Southeast University, Nanjing, PR China; 2 Robert W. Franz Cancer Research Center, Earle A. Chiles Research Institute, Providence Portland Medical Center, Portland, OR, USA

## Abstract

It is well-known that vaccines comprising of irradiated whole tumor cells or tumor-derived heat shock proteins can generate tumor-specific immune responses. In contrast, we showed recently that vaccines composed of autophagosomes (DRibbles) derived from syngeneic sarcomas could induce cross-reactive T-cell responses and cross-protection against the tumor. This unusual property of DRibbles was related to the selective recruitment of defective ribosomal products (DRiPs) and other short-lived proteins (SLiPs) into autophagosomes via sequestosome (SQSTM1, p62) mediated association of ubiquitinated SLiPs to the autophagy gene product LC3. Here, we extend our observations to mammary carcinomas from mice of different genetic background. We demonstrated that combined of intranodal administration of autologous or allogeneic DRibbles together with anti-OX40 antibody led to robust proliferation, expansion, and differentiation of memory and effector T cells. We also showed that SLiPs is an excellent source of antigen for cross-priming of CD8^+^ T-cells that recognize shared tumor antigens in the context of host MHC class I molecules. Thus, our results provide a strong basis for novel clinical trials that combine allogeneic “off-the-shelf” DRibble vaccines together with antibodies against co-stimulatory molecules.

Dr. William Coley may have attempted the first immunotherapy in cancer patients more than a century ago^1^; however, a widespread success of cancer immunotherapy was realized only recently in patients treated with antibodies against immune checkpoints. Anti-CTLA-4 and anti-PD1 antibodies have resulted in long-term disease control in patients with metastatic melanoma, non-small-cell lung cancer, and other tumor types^2^,^3^,^4^. The immune checkpoint blockade showed extraordinary advantages of tumor immunotherapy, more effective at killing cancerous tumors and cause less toxicity, lower the damage to surrounding healthy tissue and prevent debilitating side effects that are nearly unavoidable with radiation and chemotherapy^5^. Despite the long-awaited success, this revolutionary therapy is only effective in a minority of patients, seemingly in patients whose tumors are highly mutated and infiltrated with pre-existing T-cells that recognize neo-epitopes^6^,^7^,^8^. For patients whose tumors have a lower mutation burden and sparse immune infiltrate, novel strategies are needed to induce *de novo* T-cell mediated immune responses against cryptic epitopes that are ignored by the host immune system^9^. Theoretically, vaccination would be the best approach to elicit *de novo* T-cell mediated immune responses against cryptic neo-epitopes.

The successful application of cancer vaccines needs to overcome two major barriers^10^,^11^,^12^. Most previous strategies generally failed to elicit strong T-cell mediated immune responses in patients whose tumors have a low mutational burden and are poorly immunogenic^13^. Second, the immune suppressive tumor microenvironment is capable of rendering vaccine-induced effector T cells ineffective. It is thus not surprising that cancer vaccines have demonstrated little activity in the absence of strategies that effectively ameliorate the immune suppression after vaccine administration.

We hypothesized that more robust T-cell immune responses could be induced if hidden antigenic epitopes could be exposed and delivered into dendritic cells for efficient cross presentations. DRiPs contain a very large and broad spectrum of hidden epitopes including these derived from unique neo-antigens or shared tumor-associated antigens. DRiPs are not targeted by conventional cancer vaccines because they are rapidly degraded by the proteasome after their synthesis and not available for cross-presentation^14^,^15^. Recently, we have developed a novel tumor-derived autophagosome-based therapeutic vaccine (DRibbles) that could efficiently prime tumor-reactive CD8^+^ T cells via cross-presentation. Because DRiPs and other SLiPs are stabilized by proteasome inhibition, we hypothesized that DRibbles, autophagosome-containing vesicles isolated from bortezomib-treated cells, would contain SLiPs including DRiPs and thereby provide a broad spectrum of hidden epitopes including both unique neo-antigens and shared tumor-associated antigens.

DRibbles are targeted to antigen cross-presentation pathway of dendritic cells via the DC-specific receptor, CLEC9A^16^. DRibbles induced robust anti-tumor responses against established 3LL lung carcinoma when they were loaded onto DCs in the presence of IFN-γ and TLR agonist^17^. Furthermore, we showed that DRibbles from syngeneic sarcomas could prime cross-reactive T cells that recognize a panel of independently derived sarcomas. We also provided evidence that ubiquitinated SLiPs recruited by p62 sequestosome into DRibbles were critical for the priming of cross-reactive T cells against shared sarcoma antigens^18^.The novel DRibble vaccine showed the great potential to target the hidden antigenic epitopes and enhance the T-cell immune responses, but for all of that, therapeutic cancer vaccines have not been very effective when used alone in preclinical studies and clinical trials. One major hindrance could be the limited scope and insufficient magnitude of the vaccine-induced T-cell immune responses. We hypothesized that DRibble-induced T-cell expansion could be boosted by co-administration of co-stimulatory antibodies such as anti-OX40 (CD134). Anti-OX40 co-stimulation could directly stimulate CD4 and CD8 T cells and promote effector T cell expansion^19^. Base on its antitumor effects in a variety of preclinical models, anti-OX40 co-stimulatory antibody is in clinical development, a phase I clinical trial of anti-OX40 antibody therapy showed it was well-tolerated and exhibited evidence of anti-tumor activity^20^.

Here, we tested a new immunotherapy strategy that combines intranodal administration of the DRibble vaccine with an anti-OX40 co-stimulatory antibody in a murine mammary carcinoma model and found that this combination dramatically enhanced T-cell priming and anti-tumor efficacy of the DRibble vaccine. Furthermore, we investigated the ability of DRibbles from allogeneic tumor cells from mice of different genetic backgrounds to prime cross-reactive T cells and provided direct evidence that SLiPs were the source of shared tumor antigens that induced cross-reactive T cells.

## Results

### Intranodal administration of DRibbles generates superior antigen-specific T-cell activation and expansion

**Figure 1:**
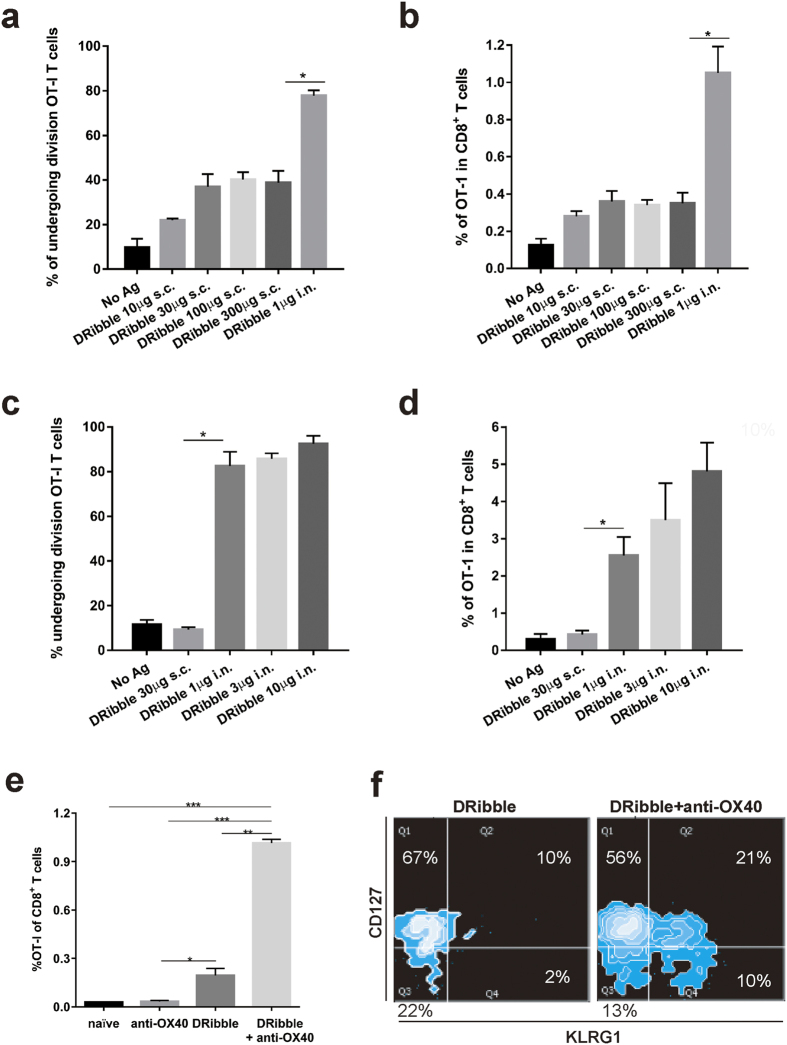
The effect of vaccine administration route and costimulation with anti-OX40 antibodies on the T-cell expansion and differentiation. To determine the most efficient route for DRibbles in the cross-priming of CD8^+^ T cells, DRibbles derived from 3LL cells expressing the OVA antigen were injected at the indicated doses into C57BL/6 mice via s.c. or i.n. injection after adoptive transfer of 1 × 10^6^ CFSE-labeled TCR transgenic Thy1.1^+^ OT-I T cells. The degree of OT-I proliferation was evaluated by staining OT-I T cells with antibodies against CD8, Thy1.1 and Vα2, flow cytometry analysis of CFSE dilution at day 5 after vaccination. (**a**) CFSE division (**b**) Frequency of OT-I T cells after s.c. injection of 10~300 μg of DRibbles. (**c**) CFSE division and (**d**) Frequency of OT-I T cells after i.n. injection of 1~10 μg DRibbles. To investigate the effect of anti-OX40 costimulation on the T-cell expansion and differentiation induced by DRibbles, naïve C57BL/6 mice were adoptively transferred with 10,000 splenocytes from Thy1.1 OT-I transgenic mice. OVA-DRibbles 10 μg were administrated into two inguinal lymph nodes and 100 μg anti-OX40 antibody was given i.p. twice 3 days apart, on the day of DRibble injection and day 3. Seven days later, spleens were harvested from control mice that received adoptive transfer of OT-I T cells alone, anti-OX40 alone, DRibble vaccination only, or a combination of DRibble vaccination and anti-OX40. (**e**) The percentage of OT-I T cells was determined by flow cytometry analysis (anti-CD8, anti-Thy1.1, Vα2). Data represent the mean and standard error of the mean from three mice per group. (**f**) Additional antibodies against CD127 and KLRG1 were used to determine the phenotypes of OT-I T cells from different mice. The plot represents a typical result from one of three mice. The experiment was repeated once and similar results were obtained. *P < 0.05; **P < 0.01; ***P < 0.001.

We have demonstrated that DRibbles isolated from a number of melanoma and lung cancer cell lines served as effective therapeutic tumor vaccines in preclinical models when given via subcutaneous administration alone or loaded onto dendritic cells^17^. To test whether i.n. administration of DRibbles without dendritic cells could lead to cross-priming of antigen-specific T cells, naïve C57BL/6 mice were adoptively transferred with CFSE-labeled naïve OT-I T cells and immunized with DRibbles derived from OVA-expressing 3LL tumor cells (OVA-DRibbles). Flow cytometry analysis of OT-I T cells in spleens from vaccinated mice showed that subcutaneously injected OVA-DRibbles were poor drivers of the OT-I T-cell division. The percentage of OT-I division and percentage of OT-I among CD8 T cells reach to a maximum level of 40% and 0.4% respectively, when the 10 to 30 μg DRibbles were administrated subcutaneously (Fig. 1a,b). No further increase of OT-I cells was observed when the amount of DRibbles were increased to 300 μg. In contrast, intranodal administration of 1 μg DRibbles resulted in division of 80% of OT-I T cells and significantly higher percentage OT-I T cells in the spleen (Fig. 1b,c). The expansion of OT-I T cells was DRibble dose dependent; when injection of DRibbles increased from 1 μg to 10 μg, a doubling of OT-I T cells was found in spleens 7-days after i.n. vaccination (from 2.5 to 5%) (Fig. 1d). When the levels of maximal levels of T-cell activation and expansion were compared, intranodal injection (10 μg) drove 20-folder higher level of T-cell division than subcutaneous injection (30 μg) (Fig. 1d). We conclude that the i.n. route is superior to the s.c. route for the direct administration of DRibble vaccines.

### Anti-OX40 co-stimulation promoted the proliferation and expansion of memory precursor (MPEC), transitional cells, short-lived memory T cells (SLEC), and memory T cells

To test the hypothesis that anti-OX40 co-stimulation could boost the DRibble-induced T-cell expansion, naïve C57BL/6 mice received a small number (1 × 10^4^) of naïve congenic Thy1.1^+^ OT-I transgenic T cells to have a similar frequency of endogenous OVA-specific T cells before they were vaccinated with OVA-DRibbles via the i.n. route. Anti-OX40 (100 μg) was given i.p. twice, on the day of vaccination and day 3 after vaccination. Seven days after vaccination, the percentage of Thy1.1^+^ OT-I T cells in the spleens of different groups of mice was determined by flow cytometry analysis using antibodies against Thy1.1 and OT-I TCR α chains. Anti-OX40 treatment led to approximately a 5-fold increase in the percentage of OT-I T cells (from 0.2% to 1.0%) compared to vaccination alone (Fig. 1e).

It has been well-documented that T-cell differentiation is coupled with T-cell division and generation of long-lasting memory T cells along with short-lived effector cells is critically important for T-cell mediated tumor regression^21^,^22^,^23^. Thus, we investigated the effect of anti-OX40 co-stimulation on T-cell differentiation via a double gating strategy with the T-cell surface markers CD127 and KLRG1. At the day of T-cell peak expansion, the majority (67%) of OT-I T cells driven by DRibble vaccination alone stained positive for CD127 and negative for KLRG1, indicating that they were mainly memory precursor effector cells (MPEC). A small number (22%) of early effector cells (EEC) that had lost CD127 but not acquired KLRG1 expression was also induced at this stage (Fig. 1f, left panel). Anti-OX40 treatment dramatically changed the memory effector composition by inducing KLRG1 expression (Fig. 1f, right panel). Although the majority of OT-I T cells in this stage were still MPEC (56%), 10% of them had lost the expression of memory T cell marker CD127 and gained the expression of KLRG1- a phenotype reflecting short-lived effector T cells (SLEC). Interestingly, approximate 20% OT-I T cells stained positive for both CD127 and KLRG1- a phenotype that has not been well-characterized and rarely seen on long-term memory T cells. This population probably represents transitional cells between MPEC to SLEC (Supplementary figure S1). Due to the limited T-cell division, DRibble vaccination alone primarily induces MEPC. The combination with anti-OX40 co-stimulation not only greatly enhanced vaccine-induced CD8^+^ T-cell proliferation but also programmed antigen-driven T cells toward both memory and effector phenotypes.

### Cross-priming of 4T1-specific T cells by DRibbles isolated from allogeneic tumor cells

**Figure 2:**
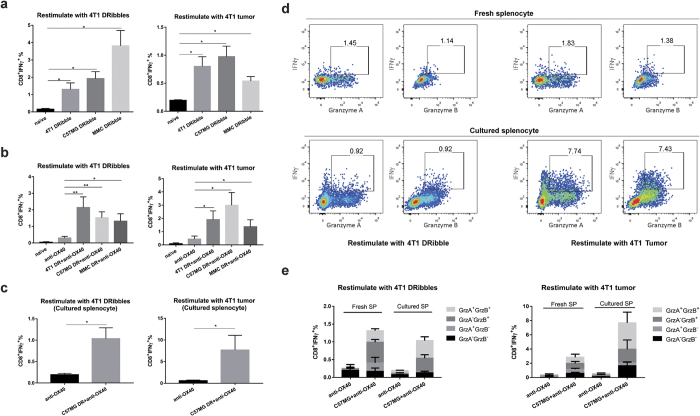
Functional analysis of tumor cross-reactive T cells elicited by DRibble vac DRibble vaccination and in combination with anti-OX40 costimulation. (**a**) Naïve BALB/c mice were vaccinated with 4T1, C57MG, and MMC DRibbles intranodally and splenocytes were isolated 7 days after vaccination and restimulated with either 4T1 DRibbles pulsed onto naïve spleen APCs (left panel) or directly with whole 4T1 tumor cells (right panel). The production of intracellular IFN-γ after restimulation was determined as described in the method section. Untreated naïve mice were used as negative controls. (**b**) Same as (**a**) with additional i.p. injection of anti-OX40 antibody at 3 and 5 after DRibble injection. Mice treated with anti-OX40 alone were included as an additional control. (**c**) Spleen cells from primed mice with C57MG DRibbles and anti-OX40 antibody were kept in culture with complete media without adding exogenous cytokine for 5 days before they were used as effector T cells for the measurement of effector functions by intracellular staining and flow cytometry analysis. (**d**,**e**) Beside the production of IFN-γ, both freshly isolated and cultured spleen CD8^+^ T cells were capable of producing granzyme A and B upon restimulation with 4T1 DRibbles or tumor cells. Data represent the mean and standard error of the mean from three or four mice per group. Independent experiments were performed two to three times. *P < 0.05; **P < 0.01.

Cross-reactivity of the immune response induced by DRibbles derived from syngeneic sarcoma cells with distinct antigenicity suggests that DRibbles could unveil cryptic tumor-specific antigens that are shared by distinct tumors with the same histology^18^. First, we sought to determine whether similar phenomena were observed when tumors of different genetic backgrounds were studied. Specifically, we asked whether DRibbles from a variety of allogeneic mammary carcinoma cell lines could induce 4T1-specific T cells in BALB/c mice. Spleens containing the DRibble-primed T cells were collected 7–9 days post intranodal injection of DRibbles and re-stimulated with either 4T1 DRibbles pulsed on naïve spleen cells or irradiated 4T1 cells. When CD8^+^ T cells from mice vaccinated with 4T1 DRibbles were re-stimulated with 4T1 DRibbles, we found that around 1.5% of primed splenic CD8^+^ T cells produced IFN-γ upon stimulation (Fig. 2a, left panel). Surprisingly, CD8^+^ T cells primed by allogeneic MMC or C57MG DRibbles also generated a comparable or higher response (2–3.5%) than CD8^+^ T cells primed by autologous 4T1 DRibbles (1–2%). However, when irradiated 4T1 tumor cells were used to re-stimulate primed effector T cells, autologous DRibbles from 4T1 tumor cells and allo-DRibbles derived from C57MG tumor cells primed a similar level of 4T1 tumor-reactive CD8^+^ effector T cells (around 0.75% IFN-γ producing CD8^+^ T cells) (Fig. 2a, right panel). Allo-DRibbles from MMC tumor cells also primed 4T1 tumor-reactive CD8^+^ T cells (around 0.5%).

Next, we also performed combinatory experiments with DRibble vaccination and anti-OX40 costimulation. Mice were vaccinated with DRibbles isolated from mouse mammary carcinoma cell lines (4T1, C57MG, and MMC) and treated with anti-OX40 on day 3 and 5 after intranodal injection of DRibble vaccines. Naïve untreated mice and mice received only anti-OX40 antibody were included as controls. Splenocytes were isolated on day 9 post vaccine injection and restimulation either with 4T1 DRibble pulsed onto naïve spleen cells as APC or 4T1 tumor cells. Anti-OX40 antibody treatment increase the background responses of spleen cells to both stimulation slightly, 0.5% CD8^+^ T cells produced IFN-γ upon *in vitro* restimulation (Fig. 2b). Approximate 2% of CD8^+^ T cells from mice received DRibble vaccines together with anti-OX40 antibodies produced IFN-γ upon restimulation with either 4T1 DRibbles pulse onto spleen APC or whole 4T1 tumor cells.

To demonstrate DRibble cross-primed T cells also exhibit effector functions other than IFN-γ, we performed experiments to determine whether cross-primed T cells could exhibit additional effect functions in addition to IFN-γ (Fig. 2c). To that end, splenocytes were isolated from mice vaccinated with C57MG DRibble plus anti-OX40 were either stimulated *ex vivo* or after culture in CM without exogenous cytokine for 5 days with 4T1-DRibbles pulsed onto spleen APC or whole 4T1 tumor cells (Fig. 2c–e). More than 70% of these IFN-γ producing CD8 T cells also produced granzyme A or B, indicating they were typical effector/memory T cells (Fig. 2d,e). A similar functional profile was observed when spleen T cells from mice received 4T1 or MMC DRibbles were analyzed (data not shown). Together, we conclude that a combination of either autologous or allogeneic DRibble vaccines and anti-OX40 costimulation is a potent strategy to induce functional 4T1 tumor-reactive CD8^+^ T cells that could mediate potent antitumor efficacy.

### Treatment of mice bearing established 4T1 tumors with intranodal administration of autologous and allogeneic DRibbles in combination with anti-OX40 co-stimulation

**Figure 3:**
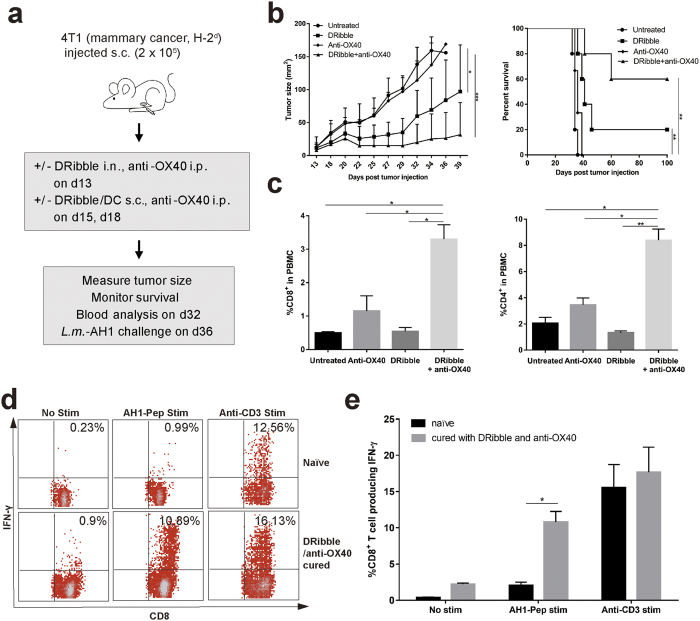
A combination of intranodal injection of 4T1 DRibbles and anti-OX40 co-stimulation induced potent anti-tumor immune responses and mediated significant tumor regression and cure of mice bearing established 4T1 tumor cells. (**a**) A schema of the immunotherapy strategy that combines intranodal vaccination with DRibbles and co-stimulation with the anti-OX40 antibody. 4T1 cancer cells were s.c. injected (20,000) into the right mammary fat pad of BALB/c mice. On day 13, mice received DRibble vaccine (20 μg, i.n.), followed by two boosts with DRibble-loaded DCs (20 μg/3 million, s.c.) on day 15 and 18. Anti-OX40 was given i.p. immediately after each DRibble vaccination at 100 μg per mouse. (**b**) DRibble vaccination combined with anti-OX40 co-stimulation led to remarkable tumor regression and cure of 60% of the treated mice. **(c**) DRibble vaccination in conjunction with anti-OX40 significantly increased the frequency of both CD4^+^ and CD8^+^ T cells in the peripheral blood mononuclear cells (PBMC) of tumor-bearing mice. Blood was collected at day 32 and PBMC were analyzed by flow cytometry to determine the percentage of CD4 and CD8 subset in total PBMC (CD45^+^) after purification through a density gradient. Data represent the mean and standard error from three mice per group. **(d,e)** DRibble/anti-OX40 combination vaccine cured mice and control naïve mice were challenged with 100,000 Delta-actA-Lm-AH1-A5 i.v. Splenocytes were collected and re-stimulated with AH1 peptide a week later. ICS of IFN-γ producing CD8^+^ T cells was performed. Independent experiments were performed three times. *P < 0.05; **P < 0.01.

Next, we examined the efficacy of DRibble vaccine with or without anti-OX40 co-stimulation in the orthotopic 4T1 mammary cancer model. The highly metastatic 4T1 cancer cells were injected directly into the mammary pads and metastases could be found in distal organs such as the brain, lung, liver, and bone 7–10 days after tumor injection^24^. To demonstrate the antitumor efficacy in mice with advanced 4T1 tumor burden, 4T1 tumors were allowed to grow for 13 days before initiation of therapy, at which time the mean diameter of tumors reached 3–5 millimeters (Fig. 3a). Mice bearing 13-day 4T1 tumors were divided into different treatment groups or left untreated. Approximately 35 days after tumor inoculation, all untreated control mice had to be euthanized due to large tumor burdens; mice treated with DRibble vaccination alone or anti-OX40 alone exhibited a limited tumor control (Fig. 3b). The combination of DRibble vaccination and anti-OX40 treatment showed a dramatic synergistic effect on the survival of mice. Not only tumor growth was significantly delayed, but also tumors were eradicated in 60% of mice. Consequently, the median survival was dramatically improved as compared to control mice.

To correlate the therapeutic efficacy of combination therapy with immune changes, we first determined the percentage of total CD8^+^ and CD4^+^ T cells in the peripheral blood of mice treated with DRibble vaccine, anti-OX40 antibody, or a combination of these two agents. Interestingly, we found that the percentage of blood CD8^+^ and CD4^+^ T cells was greatly increased in mice treated with the anti-OX40 antibody; no significant change was found in mice only received DRibble vaccine. However, a combination of DRibble vaccination and anti-OX40 antibody further increased the percentage of blood CD8^+^ and CD4^+^ T cells (Fig. 3c). Both anti-OX40 costimulations could induce antigen-independent proliferation and affect the effector/memory T-cell trafficking into the blood from lymphoid organs, it is expected that both antigen-dependent and independent proliferation and trafficking contributed to the much-heightened blood CD8^+^ and CD4^+^ T cells in mice treated with DRibble vaccine and anti-OX40 antibody.

To detect tumor antigen-specific CD8^+^ T cell responses, mice that were cured by the DRibble and anti-OX40 therapy were challenged with *Listeria monocytogenes* expressing the AH1 epitope derived from gp70 antigen, which is an endogenous antigen expressed by 4T1 tumor cells. We measured the ability of splenic CD8^+^ T cells to produce IFN-γ in response to AH1 peptide *ex vivo* a week later (Fig. 3d and e). More than 10% of splenic CD8^+^ T cells in mice cured by DRibble/OX40 combination therapy produced IFN-γ when re-stimulated with AH1 peptide. However, few if any CD8^+^ T cells from the untreated mice responded to the re-stimulation. Taken together, these data suggest that autologous DRibbles from 4T1 combined with anti-OX40 can be used to treat 4T1 breast tumor effectively and 4T1 DRibble vaccine induced AH1-specific T cells.

**Figure 4:**
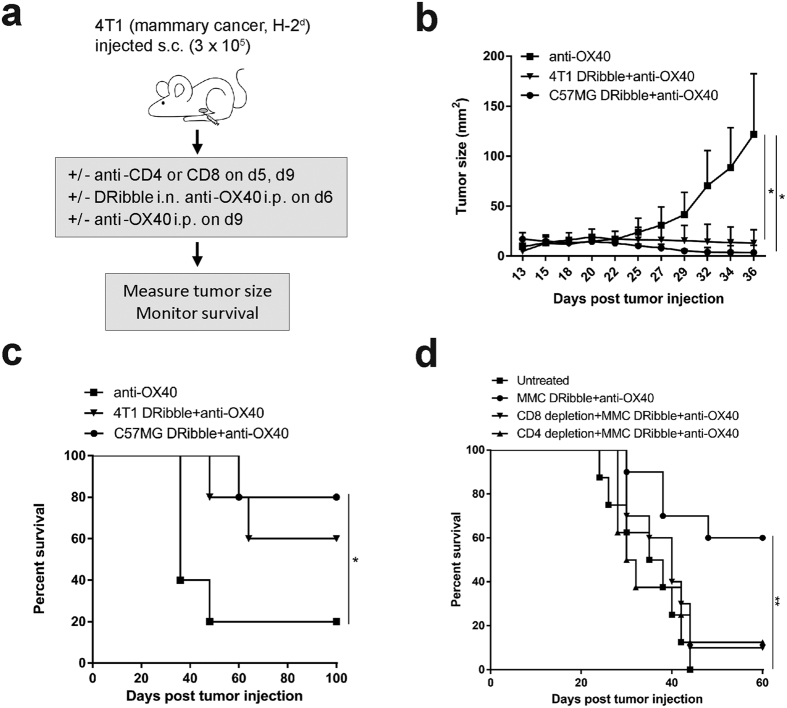
The allogeneic DRibble vaccination in combination with anti-OX40 antibody mediated a potent antitumor response and required both CD4^+^ and CD8^+^ T-cell subsets for its efficacy. (**a**) To evaluate whether allo-DRibbles can confer anti-tumor immunity against 4T1, we administrated allo-DRibbles from C57MG **(b,c)** or MMC **(d)** mammary carcinoma cells. BALB/c mice bearing 13-day 4T1 tumors were established as in Fig 2 and treated with DRibbles from C57MG tumors, 4T1 tumors, followed by two boosts with DC s.c. at 2 day interval. Anti-OX40 (100 μg) was injected i.p along with each immunization. C57MG allo-DRibbles resulted in 80% cure rate of BALB/c mice bearing 4T1 tumors. To determine the role of CD4^+^ or CD8^+^ T cells for the anti-tumor efficacy, 4T1 tumor cells (30,000) were injected into the right mammary pads of BALB/c mice. At day 5, mice with palpable tumors were divided into four experimental groups: untreated, DRibble vaccination and anti-OX40, DRibble vaccination and anti-OX40 with CD8 depletion, DRibble and anti-OX40 with CD4 depletion. Anti-CD8 (100 μg) and anti-CD4 (200 μg) was given twice i.p. to deplete CD8^+^ and CD4^+^ T cells respectively on day 5, 9. Mice received DRibbles (i.n., 20 μg) from MMC tumors on day 6. Anti-OX40 (100 μg) was given i.p. at day 6 and 9. Tumor growth was measured every other day on weekdays starting from day 10 (**c**), and mouse survival was monitored (**d**). Independent experiments were performed two times. *P < 0.05; **P < 0.01.

Next, we investigated the efficacy of allogeneic DRibbles from established mammary carcinoma cell lines. To this end, we tested the ability of allo-DRibbles to prime therapeutic immune responses against 4T1 tumors and compared their relative effectiveness to autologous 4T1 DRibbles in BALB/c mice bearing 13-day 4T1 tumors (Fig. 4a). We found that vaccination with allo-DRibbles from C57MG tumor cells led to substantial tumor regression of 4T1 tumors (Fig. 4b). Notably, vaccination with C57MG allo-DRibbles in combination with anti-OX40 antibody resulted in 80% mice being cured of established 4T1 tumors, significantly higher survival rate than vaccination with 4T1 autologous DRibbles (50%) (Fig. 4c). This indicates that allo-DRibbles may be useful for the treatment of mammary tumors effectively. A similar result was obtained using allo-DRibbles derived from MMC tumor cells; 60% of BALB/c mice bearing established 4T1 tumors were cured by treatment with MMC DRibbles plus anti-OX40 antibody administration (Fig. 4d).

To evaluate the role of CD8^+^ and CD4^+^ T cells in tumor regression, anti-CD8 or anti-CD4 antibodies were used to deplete corresponding T-cell subsets one day before vaccination and every 3–4 days thereafter for 3 injections. Depletion of either CD8^+^ T cells or CD4^+^ T cells nearly eliminated the anti-tumor effect of the combinational treatment of MMC DRibbles and anti-OX40 antibody (Fig. 4d). Our results indicate that both CD8^+^ and CD4^+^ T cells are required for the anti-tumor immunity induced by DRibble and anti-OX40 combination immunotherapy. The requirement of both subsets of T cells is not unique to MMC DRibble vaccine. Our ongoing studies with other tumor models also showed that the same requirement for both CD4 and CD8 T cells for the antitumor effect of DRibble vaccines. We observed a similar requirement for CD4 and CD8 T cells in experiments with Line-1 lung and SCC-VII oral squamous cell carcinoma (manuscripts in preparation).

### SLiPs isolated from tumor cells efficiently stimulate antigen-specific naïve T cells *in vitro*

In eukaryotic cells, proteasome inhibition results in shunting of ubiquitinated SLiPs into autophagosomes with the help of ubiquitin-binding receptors p62 and NBR1^25^,^26^,^27^. Recently, Sims *et al*. created a synthetic Ub-binding protein, Vx3(A7), which contains multiple tandem ubiquitin-interacting motifs (tUIMs) with structured linker regions that preferentially bind to Lys63-polyUb^28^. Since DRibbles contain Lys63-polyUb and our previous publication suggested a critical role for p62 in the accumulation of SLiPs with Lys63-polyUb and immunogenicity of DRibbles^18^, we used Vx3(A7) to isolate ubiquitinated SLiPs from tumor cells. We produced a GFP fusion protein of Vx3(A7) and GFP (Vx3GFP) to facilitate both bacterial expressions, purification, and functional delivery of isolated polyUb conjugated proteins into antigen presenting cells (Supplementary figure S2A). After treatment with bortezomib, SLiPs could be isolated from lysates of whole tumor cells by Vx3GFP (Supplementary figure S2B).

OVA-expressing tumor cells and OT-I TCR transgenic T cells were first used to test whether SLiPs could stimulate naïve CD8^+^ OT-I T cells *in vitro*. SLiPs from B78H1 melanoma cells stably expressed the short-lived GFP-OVA fusion protein (B78H1-OVA) and native GFP (B78H1-GFP). B78H1 melanoma cells were used here because they do not express classical MHC-I molecules and lack the TAP2 gene^29^,^30^. The mutu-1940 DC cell line was used as the APC and alumina nanoparticles were used to facilitate intracellular delivery of isolated SLiPs^31^. Mutu-1940 DC was an established cell line from CD1c-SV40 large T antigen transgenic C57BL/6 mice^32^ and it was shown to closely resemble endogenous CD8^+^ conventional DCs (cDC). When CFSE labeled naïve OT-I T cells were stimulated by SLiPs-loaded mutu-1940 DCs, OVA-specific T-cell proliferation was observed when SLiPs was purified from B78H1-OVA but not B78H1-GFP tumor cells. In addition, DCs pulsed with SLiPs from normal livers also failed to stimulate OT-I T cells (Supplementary figure S3). These results showed that isolated SLiPs, when loaded onto DCs, could be a rich source of antigens that are capable of driving antigen-specific CD8^+^ T-cell proliferation.

**Figure 5:**
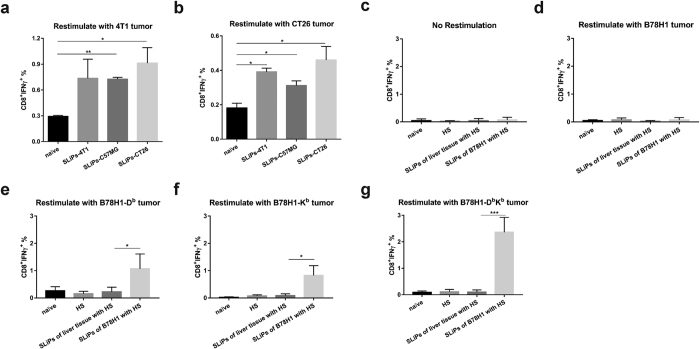
Cross-priming of tumor cross-reactive T cells using SLiPs isolated from allogeneic or MHC class Ia deficient tumor cells. To evaluate whether isolated ubiquitinated SLiPs from allogeneic mammary cancer cells (C57MG) or syngeneic colon cancer cells (CT26), we administered isolated SLiPs from tumor cells via the i.n. route into BALB/c mice. Seven days later, mice were sacrificed and splenocytes were restimulated with 4T1 cells or CT26 cells. Intracellular IFN-γ staining was performed to determine the frequency of tumor-reactive CD8^+^ T cells induced by vaccination with SLiPs. (**a**) CD8^+^ T cell responses to 4T1 cells. (**b**) CD8^+^ T cell responses to CT26 cells. To evaluate whether SLiPs isolated from tumor cells could induce tumor-reactive T cells, we vaccinated C57BL/6 mice with SLiPs from B78H1 melanoma cells together with alumina nanoparticles (HS) as the adjuvant. Mice vaccinated with HS adjuvant alone or SLiPs from normal liver tissue plus HS were included as controls. Seven days later, mice were sacrificed and splenocytes were restimulated with irradiated B78H1 (**d**), B78H1-D^b^ (**e**), B78H1-K^b^ (**f**) and B78H1-D^b^K^b^ (**g**) tumor cells, CM as a negative control (**c**). Intracellular IFN-γ staining was performed to determine the frequency of tumor-reactive CD8^+^ T cells induced by vaccination with SLiPs. Data represent the mean and standard error of the mean from five mice per group. Independent experiments were performed three times. *P < 0.05; **P < 0.01; ***P < 0.001.

Beside the gp70 antigen, we currently do not know the identities of shared tumor-associated antigens expressed by 4T1, MMC, or C57MG mammary tumor cells. However, our published work indicated that SLiPs accumulated in DRibbles was critical for cross-protection induced by sarcoma DRibble in C57B6 mice^18^. To directly demonstrated whether SLiPs could induce cross-reactive T cells, we isolate SLiPs from 4T1, C57MG (H-2^b^), and CT26 tumor cells and examined their ability to prime MHC I-restricted T cells that recognize 4T1 or CT26 tumor cells in BALB/c mice. BALB/c mice were vaccinated with isolated SLiPs via the i.n. route without adjuvant. Primed splenocytes were collected and re-stimulated with irradiated autologous 4T1 or syngeneic CT26 colon tumor cells. Consistent with the notion that SLiPs are sources of shared antigens, a similar percentage of primed CD8^+^ T cells primed by either one of three SLiPs responded to the 4T1 tumor re-stimulation *in vitro* (Fig. 5a). Surprisingly, primed T cells were also responded to restimulation with irradiated CT26 colon tumor cells (Fig. 5b). These data further suggest that tumor-derived SLiPs can cross-prime tumor-reactive T cells to recognize not only breast cancer cells but also colon cancer cells.

### Classical MHC I restricted tumor-reactive T cells could be induced *in vivo* by immunization with purified SLiPs

To further support the hypothesis that isolated ubiquitinated SLiPs are a good source of antigens for cross-priming of classical MHC-I restricted tumor-reactive T cells *in vivo*, we turn to B78H1 melanoma cells, which are deficient of MHC-Ia molecules (H-2D^b^ or H-2K^b^). B78H1-D^b^, B78H1-K^b^, B78H1-D^b^K^b^ stable cell lines that had been transfected to stably express H-2 molecules were used to determine whether tumor-reactive T cells induced by SLiPs are restricted by these MHC-I molecules. To this end, C57BL/6 mice were vaccinated with isolated SLiPs derived B78H1 melanoma cells together with alumina adjuvant. Seven days after vaccination with SLiPs, splenocytes were restimulated with irradiated B78H1, B78H1-D^b^, B78H1-K^b^, and B78H1-D^b^K^b^ cells to determine whether T cells primed by isolated SLiPs can recognize B78H1 tumor cells by intracellular IFN-γ release assay and flow cytometry analysis. B78H1 tumor cells that express H-2D^b^, K^b^, or both, but not parental B78H1 tumor cells, could stimulate T cells from mice primed by SLiPs (Fig. 5c–g). As additional controls, mice were also vaccinated with SLiPs derived from normal livers or alumina adjuvant alone. T cells from naïve mice, mice received normal kidney SLiPs, or mice received adjuvant alone did not respond to the *in vitro* stimulation with tumor cells. These data strongly clearly showed that ubiquitinated SLiPs isolated from MHC-Ia deficient tumor cells could cross-prime MHC-Ia restricted CD8^+^ T cells *in vivo*.

## Discussion

We have shown that intranodal administration of DRibbles combined with intraperitoneal anti-OX40 antibody induced antigen-specific T-cell activation and expansion and tumor regression in mice bearing established 4T1 tumors. Most interestingly, we found that DRibbles isolated from allogeneic cells induced cross-reactive T-cells immune responses led to the cure of mice bearing established 4T1 tumors, with efficacy similar to autologous DRibble vaccine. We also provided evidence that as ubiquitinated SLiPs when purified from tumor cells, in fact, was an excellent source of shared tumor antigens to stimulate cross-reactive T cells. In addition, SLiPs derived from allogeneic breast cancer cells or syngeneic colon cancer cells could cross-prime tumor-reactive T cells to recognize 4T1 and CT26.

Antigens exposure at different locations may interact with distinct APCs and may be presented to T cells with differential efficiency^33^. Cancer vaccines can be administered by a variety of routes: subcutaneous, dermal, intravenous, intratumoral, and intranodal. The intranodal route of vaccination with DNA, RNA or DC loaded with tumor antigenic peptides or tumor lysates, has been shown to be superior to other routes in eliciting CD8^+^ T cell responses in both preclinical and clinical settings^34^,^35^,^36^,^37^. Our results confirmed that intranodal vaccination was superior to subcutaneous administration. In addition, DRibble needed to be loaded onto dendritic cells to induce anti-tumor effect by subcutaneous administration^17^; however, when DRibbles were delivered directly to a lymph node, exogenous DC administration was not required.

Many published data including those from our recurrence study showed that antibodies targeting co-stimulatory molecules expressed on T cells may play an important role in improving the anti-tumor efficacy of the therapeutic cancer vaccines. An agonist anti-OX40 antibody can augment the generation of both memory and effector T cells and enhance the function of vaccine-primed T cells. We showed that anti-OX40 antibody induced the KLRG1 expression on antigen-specific T cells upon antigen stimulation and generation of both SLEC and translational cells that in an intermediate stage from MPEC to SLEC (Fig. 1f). A similar but less profound induction of KLRG1^+^ SLEC was also noticed in mice during the early phase of an adenovirus infection plus anti-OX40 co-stimulation by a recent publication^38^. However, an endogenous OX40 signaling was shown to be dispensable for the generation of KLRG1^hi^ SLEC but was essential for the maintenance of KLRG1^low^ MPECs during infection with Listeria monocytogenes^39^. This apparent discrepancy is likely due to the difference in TLR signaling and the signaling strength delivered by endogenous OX40 L and exogenous anti-OX40 antibody. The efficacy of anti-OX40 is likely to be improved by combination with other therapies such as radiation and chemotherapy or other immune approaches. In this study, we showed that combination of anti-OX40 with DRibble intranodal vaccination improved the efficacy of anti-OX40 alone. Beside its role in T-cell proliferation and differentiation, anti-OX40 was also shown to reduce the number and function of tumor-associated T_reg_ cells and MDSC and contribute to its antitumor efficacy^40^,^41^.

Together with our previous results with 3LL lung tumors, the successful treatment of 4T1 mammary cancer by vaccination with autologous 4T1 derived-DRibbles demonstrated the efficacy of DRibble vaccination as a proof-of-principle in an autologous setting. Independent generated MCA-induced sarcomas in genetically identical mice exhibited similar morphology and growth characteristics but unique immunogenicity vaccinated mice could only resist a challenge with the immunizing tumor but not other syngeneic MCA-induced sarcomas^42^,^43^. Surprisingly, our previous studies discovered that DRibble vaccination led to immunity against not only the homologous sarcoma but also other independently derived MCA-induced sarcomas. DRibbles vaccination resulted in cross-protection in 8 of the 9 combinations tested, whereas the whole-cell vaccine provided cross-protection in 0 of 9 combinations tested^18^. These results suggested that DRibbles contained shared tumor epitopes derived from MCA tumors, which could protect mice from both homologous sarcoma and other MCA-induced syngeneic sarcomas. Here we showed that DRibbles prepared from mammary cancer cell lines from either BALB/c, FVB or C57BL/6 mice could elicit robust cross-reactive T-cell immune responses that mediated regression long-established 4T1 tumors. Therefore, DRibbles derived from tumor cell lines of the same histology appeared to share tumor antigens regardless of MHC. Although our vaccines were derived from allogeneic tumor cell lines (C57MG from C57B6 mice or MMC from FVB mice), the tumor cells (4T1) used to create the model are syngeneic to host mice (BALB/c). When BALB/c mice bearing syngeneic 4T1 tumors were treated with allo-DRibbles derived from C57MG or MMC tumor cells, immune responses against allo-MHC (H-2^b^ or H-2^q^) or other alloantigens could be induced. However, it is extremely unlikely the immune responses directed to alloantigens could be directly involved in our setting. It is because both host cells (expressing H-2^d^) or target tumor cells 4T1 (expressing H-2^d^) do not express allo-MHC or other alloantigens. Even such allo responses were induced and lead to rejection of allo-skin grafts, these responses are not MHC compatible with either host tissues or 4T1 tumors. One possible explanation for the increased responses from MMC DRibble-immunized mice is that MMC DRibbles induced antibodies against alloantigens expressed on MMC DRibbles when BALB/c mice were vaccinated with MMC DRibbles and these antibodies could indirectly increase the efficiency of cross-presentation. Our preliminary data did support this hypothesis. We are working on characterization of these alloantibodies and alloantigens they recognize. Our ongoing studies are designed to investigate the mechanisms by which antibodies against alloantigens in DRibbles enhance the cross-presentation. Because DRibbles contain self-antigens, one concern of complex vaccines of this type is whether vaccination with DRibbles could result in an autoimmune reaction. Both preclinical and clinical studies indicated that this risk of autoimmune reaction is very low in mice and cancer patients. Although careful pathology in mice needs to be done, there is no obvious sign of autoimmune pathology in 4T1 tumor-bearing mice cured by DRibble vaccines. In addition, we are conducting a phase I/II clinical trials in patients with NSCLC with allo-DRibbles. So far, we have performed multiple DRibble vaccination (up to 8 doses) in more than 10 patients and did not observe major toxicities related to allo-DRibble vaccines.

The generation of DRibbles employs bortezomib to block proteasome degradation, and ammonium chloride to inhibit degradation of autophagic cargo inside the autolysosome (Supplementary figure S4). Because DRibbles derived from cells that lack ubiquitinated SLiPs had diminished the capacity to stimulate antigen-specific T cells and failed to protect hosts from MCA tumor challenge, it is likely that SLiPs comprise the critical antigens that induce antigen-specific antitumor immune responses shared by tumors with the same histological type. This notion is strongly supported by our results showing that SLiPs isolated from tumor cells could cross-prime MHC-Ia restricted CD8^+^ T cells *in vivo*. Similar to DRibbles, SLiPs isolated from both allogeneic and syngeneic breast cancer cells could induce anti-4T1 tumor immune responses (Fig. 5). Of note, SLiPs isolated from syngeneic CT26 colon cancer cells also primed anti-4T1 tumor immune responses. One potential shared antigen is the gp70 envelope protein encoded by an endogenous retrovirus and expressed by both CT26 and 4T1. In fact, we did observe that 4T1 DRibbles elicited anti-gp70 T cells after intranodal immunization (Fig. 3).

Previously, we found that heat shock proteins including HSP90, HSP96 (gp96), and HSP70, and calreticulin are abundant in DRibbles^17^. Heat shock proteins have been shown to play a critical role in preserving the tumor-specific antigen repertoire and augmenting cross-priming of tumor-specific T cells. Pioneering work from Srivastava *et al* elegantly showed that gp96 isolated from tumor cells, which contains epitopes derived tumor-specific mutations, could elicit tumor-specific immune responses that were unique to the tumor cells from which the gp96 proteins were isolated^44^. Subsequent publications extended their observations to other HSPs^45^,^46^. Except for a few epitopes from viral or model antigens, the tumor-specific epitopes, like the shared epitopes in SLiPs we proposed here, remain as a mystery^47^. Of note, results from Podack’s group indicated that gp96 could also present shared epitopes when gp96 was fused with IgG Fc and secreted from allogeneic tumor cells^48^. Thus, it is reasonable to propose that HSPs and ubiquitinated SLiPs in DRibbles may represent two different pools of antigens- we hypothesized that HSPs purified from tumor cells contain tumor-specific antigens that are not shared among tumor cells, and therefore would be expected to provide protection only from the same tumor challenges; whereas SLiPs provide shared antigens and can elicit cross-protection against challenge with different types of tumors, including allogeneic tumor cells. DRibble vaccines, which contain both HSPs and ubiquitinated SLiPs, could induce both tumor specific protection and cross-protection and therefore would be an ideal partner for immune checkpoint blockade and co-stimulatory antibodies.

In summary, our work reveals that the combination of intranodal administration of DRibb-les derived from either autologous or allogeneic mammary cancer and anti-OX40 co-stimulation could induce cross-reactive T-cell immune responses and tumor regression in mice bearing established 4T1 tumors. These findings imply that allo-DRibbles derived from established tumor cell lines may be packaged as an “off-the-shelf” allogeneic vaccines for the treatment of patients with breast cancer. The allo-DRibbles will have advantages in term of production, storage, and quality control over autologous DRibbles.

## Method and Materials

### Mice and Cell lines

Six~eight-week-old female BALB/c mice and C57BL/6 mice were purchased from Harlan Laboratories or Jackson Laboratory. OT-I breeders with transgenic T cell receptors (TCR) that recognize the H-2K^b^-restricted OVA_257–264_ peptide were purchased from the Jackson Laboratory. All mice were maintained and used in accordance with the animal protocol approved by the Earle A. Chiles Research Institute Animal Care and Use Committee.

Murine mammary carcinoma cell lines used in this study were 4T1 from BALB/c mice, C57MG from C57BL/6 mice, and MMC from FVB mice. 4T1 was kindly provided by Dr. Fred Miller (Michigan Cancer Foundation, Detroit, MI), C57MG was provided by Dr. Pinku Mukherjee (University of North Carolina at Charlotte, Charlotte, NC), and MMC was provided by Dr. Emmanuel T. Akporiaye (Earle A. Chiles Research Institute, Portland, OR). CT26 colon cancer cells were from BALB/c. The B78H1 cell line is a MHC-I-deficient subclone of B16 melanoma; B78H1-D^b^, B78H1-K^b^, B78H1-D^b^K^b^ stable cell lines were transfected with plasmids expressing MHC-Ia molecules, these B78H1 cell lines and CT26 were kindly provided by Hyam I. Levitsky (Johns Hopkins University, Baltimore, MD); B78H1-R-OVA-GFP and B78H1-GFP stable cell lines were transfected with plasmids expressing short-lived GFP-R-OVA fusion protein or GFP alone^16^. Mutu-1940 DCs from C57BL/6 mice were kindly provided by Dr. Hans Acha-Orbea (University of Lausanne, Epalinges, Switzerland)^32^. *Listeria mono cytogenes* that express the endogenous 4T1 tumor antigen AH1 (Delta-actA-Lm-AH1-A5) were kindly provided by Dr. Keith Bahjat (Earle A. Chiles Research Institute, Portland, OR). These cells have not undergone further testing.

DCs were generated from bone marrow cells as described^49^. Briefly, bone marrow cells (1 × 10^6^ cells/mL) were cultured in RPMI completed media supplemented with 20 ng/ml GM-CSF (Peprotech) and 70 μM beta-2-mercaptoethanol (Sigma). On day 6, the non-adherent and loosely adherent cells were collected, typically 70~90% cells were CD11c positive.

### Preparation of tumor-derived DRibbles

Tumor cells grown to 90% confluence were treated with bortezomib (100 nM) and NH_4_Cl (20 mM) for 24–48 hours. The cells and culture media were collected and spun at 1000 rpm for 10 min. Cells were washed twice with PBS containing 5 mM EDTA to dissociate DRibbles from the cell surface. The supernatant containing the crude DRibbles was centrifuged at 7500 rpm for 15 min and the DRibble pellet was resuspended in PBS and frozen in- 80 °C before use. Protein concentration was measured by BCA assay (Thermo Scientific).

### Intranodal injection of DRibbles

For intranodal immunizations, mice were anesthetized with isoflurane vaporized in 100% oxygen and transferred to a nose cone for continued anesthetic delivery. After both inguinal lymph nodes were surgically exposed, 10 μL DRibbles were injected into each node using a 30-unit insulin syringe with 30guage needle (BD). The wound was closed with small titanium ligating clips (Horizons).

### Flow cytometry analysis

Fluorescently labeled antibodies were purchased from Biolegend (CD127-Alexa Fluor 488, KLRG1-APC, Thy1.1-Pacific Blue, Thy1.2-Pacific Blue, CD4-FITC, CD8-PE, IFN-γ-APC, CD45-APC, Vα2-APC). Dead cells were excluded using the fixable aqua dead cell stain kit (Invitrogen). Brefeldin A (10 μg/ml, Sigma) was used to stop Golgi transportation of intracellular cytokines. Data were acquired using BD FACS Calibur or LSR-II and analyzed using FlowJo software (Tree Star).

### Tumor immunotherapy

Orthotopic breast cancer model was generated by injecting of 4T1 tumor cells (3 × 10^4^) subcutaneously (s.c.) into the right mammary fat pad of female BALB/c mice. For therapy, mice were immunized i.n. with 4T1 DRibbles (20 μg) on day 13, followed by two boosts with DRibble-loaded DCs (20 μg DRibbles/3 × 10^6^ DCs; s.c.) at two-day intervals. Anti-OX40 was administrated i.p. (100 μg) immediately after each vaccination. For experiments with allogeneic DRibbles, mice with palpable tumors were given DRibbles derived from MMC tumor cells (20 μg; i.n.) at day 6, followed by two i.p. injections of anti-OX40 (100 μg) on days 6 and 9. To deplete CD8^+^ and CD4^+^ T cells, designated mice received anti-CD8 (100 μg) and anti-CD4 (200 μg) respectively on day 5, 24 hours before DRibble injection. Tumor size was measured every other day on weekdays. Survival of mice was monitored.

### Expression and purification of polyUb-binding protein Vx3GFP

Recombinant Vx3GFP fusion protein with a His_6_ tag was expressed in LPS deficient BL21(DE3) clear cells (Lucigen). The expression vector, pET28a-Vx3(A7), was kindly provided by Dr. Robert E. Cohen^28^. Bacteria expressing Vx3GFP was pelleted and subjected to direct isolation by Three-Phase-Partitioning (TPP)^50^. The lower aqueous layer was diluted with TE buffer (1:1) and loaded onto a HisTrap excel chromatography column (GE). The column was first washed with Tris-NaCl buffer solution (20 mM Tris-Cl, 300 mM NaCl) with 20 mM imidazole, pH8.0), then eluted with Tris-NaCl buffer with 250 mM imidazole. Purification was performed with the ÄKTA pure chromatography system (GE).

### Isolation of tumor-derived ubiquitinated SLiPs

After treatment with bortezomib for 16–24 hours, tumor cells were collected and washed with PBS twice and resuspended in PBS. After three freeze-thaw cycles, cells were lysed and centrifuged at 12,000 rpm for 5 minutes; the supernatant was saved as freeze-thaw lysate. Vx3GFP was added to freeze-thaw lysates of tumor or normal liver tissue and incubated overnight at 4 °C. The mix was transferred into Ni-Sepharose excel resin and incubated overnight at 4 °C. After centrifugation, the unbound lysate was saved as flow through, the Ni-Sepharose excel resin was washed with Tris-NaCl buffer, then eluted with Tris-NaCl buffer with 250 mM imidazole. The eluate was dialyzed into PBS and saved as concentrated ubiquitinated SLiPs.

### Statistical methods

Statistical analyses were performed using a two-tailed Student t test or two-way ANOVA (GraphPad Prism). Error bars denote mean ± SD. A P value of less than 0.05 was considered to be statistically significant (*P < 0.05; **P < 0.01; ***P < 0.001).

## Additional Information

**How to cite this article**: Yu, G. *et al*. Combinational Immunotherapy with Allo-DRibble Vaccines and Anti-OX40 Co-Stimulation Leads to Generation of Cross-Reactive Effector T Cells and Tumor Regression. *Sci. Rep*. **6**, 37558; doi: 10.1038/srep37558 (2016).

**Publisher’s note:** Springer Nature remains neutral with regard to jurisdictional claims in published maps and institutional affiliations.

## Supplementary Material

Supplementary Information
