# Supplementary material for: Combinational Immunotherapy with Allo-DRibble Vaccines and Anti-OX40 Co-Stimulation Leads to Generation of Cross-Reactive Effector T Cells and Tumor Regression
Source: Sci Rep. 2016 Nov 22;6:37558. doi: 10.1038/srep37558 (PMC5118714; doi:10.1038/srep37558)

Combinational Immunotherapy with Allo-DRibble Vaccines and Anti-OX40 Co-Stimulation Leads to Generation of Cross-Reactive Effector T Cells and Tumor Regression

Guangjie Yu[1,2*], Yuhuan Li[2*], Zhihua Cui[2], Nicholas P. Morris[2], Andrew D. Weinberg[2], Bernard A. Fox[2], Walter J. Urba[2], Lixin Wang[1], Hong-Ming Hu[2]

[1]School of Medicine, Southeast University, Nanjing, PR China [2]Robert W. Franz Cancer Research Center, Earle A. Chiles Research Institute, Providence Portland Medical Center, Portland, OR, USA

Keywords: Allo-DRibble vaccines, anti-OX40, SLiPs, Cancer vaccines, Immune responses to cancer

Corresponding Authors: Lixin Wang, 87 Dingjiaqiao St., Nanjing, Jiangsu, PR China, 210009. Phone: 86-25-83272503, Fax: 86-25-83324887. Hong-Ming Hu, 2N81 North Pavilion, 4805 N.E. Glisan St., Portland, OR 97213. Phone: 1-503-215-6531, Fax: 1-503-215-6841. E-mail: lxwang@seu.edu.cn; hongming.hu@providence.org.

*These authors contributed equally to this work.

Other Authors:

Guangjie Yu: guangjie1113@gmail.com Yuhuan Li: yuhuan.li@providence.org

Zhihua Cui: zhihua.cui@providence.org Nicholas P. Morris: nicholas.morris@providence.org

Andrew D. Weinberg: andrew.weinberg@providence.org

Bernard A. Fox: bfox@providence.org Walter J. Urba: walter.urba@providence.org

Supplementary data

Figure S1 Anti-OX40 co-stimulation promoted the proliferation and expansion of memory precursor (MPEC), transitional cells, short-lived memory T cells (SLEC), and memory T cells.

Figure S2 Purification of DRiPs from tumor cells with a Vx3GFP fusion protein.

(**A**) A schematic representation of Vx3GFP, a fusion protein comprising an engineered, novel, tandem ubiquitin-interacting motifs (tUIMs-Vx3) and GFP, binds polyubiquitinated proteins with high affinity and selectivity toward lys63 linkage. (**B**) Efficient isolation of polyubiquitinated proteins (DRiPs) from lysate of tumor cells that were pretreated with a proteasome inhibitor. 10, 30, 100μg Vx3GFP protein were added to C57MG lysate and DRiPs were pulled down with Ni-Sepharose excel resin. The flow through fraction was saved and DRiPs binding the resin were washed with Tris-NaCl buffer containing 5mM imidazole. Bound DRiPs were eluted with Tris-NaCl buffer containing 250mM imidazole. Samples from input, flow through, and elution were separated by SDS-PAGE and purification of DRiPs was demonstrated by western blot analysis with rabbit anti-ubiquitin antibody (1:2,000; Sigma).

Figure S3 Isolated DRiPs stimulate antigen-specific T-cell proliferation efficiently when loaded onto dendritic cells.

To determine whether they could stimulate antigen-specific T cell proliferation, we isolated DRiPs from B78H1 cells that expressed R-GFP-OVA protein after pretreatment with bortezomib. DRiPs from B78H1 cells that express GFP, but not OVA, and liver tissues were included as specificity

controls. Mutu-1940 DCs were loaded with aluminum nanoparticles plus the indicated amount of DRiPs. CFSE-labeled OT-I transgenic T cells were added after a 6-hour incubation. T-cell activation was assessed by flow cytometry analysis of CFSE dilution of labeled T cells at day 6. Mutu-1940 DCs pulsed with antigen-expressing ubiquitinated DRiPs were efficient stimulators of OT-I $CD8^+$ T cells. Ubiquitinated DRiPs from normal liver tissue or antigen-free B78H1 cells barely stimulated OT-I $CD8^+$ T cells. *, $P < 0.05$; **, $P < 0.01$.

Figure S4 Schematic illustration of a possible pathway for the selective accumulation of DRiPs and generation of DRibbles.

Supplementary Figure 1

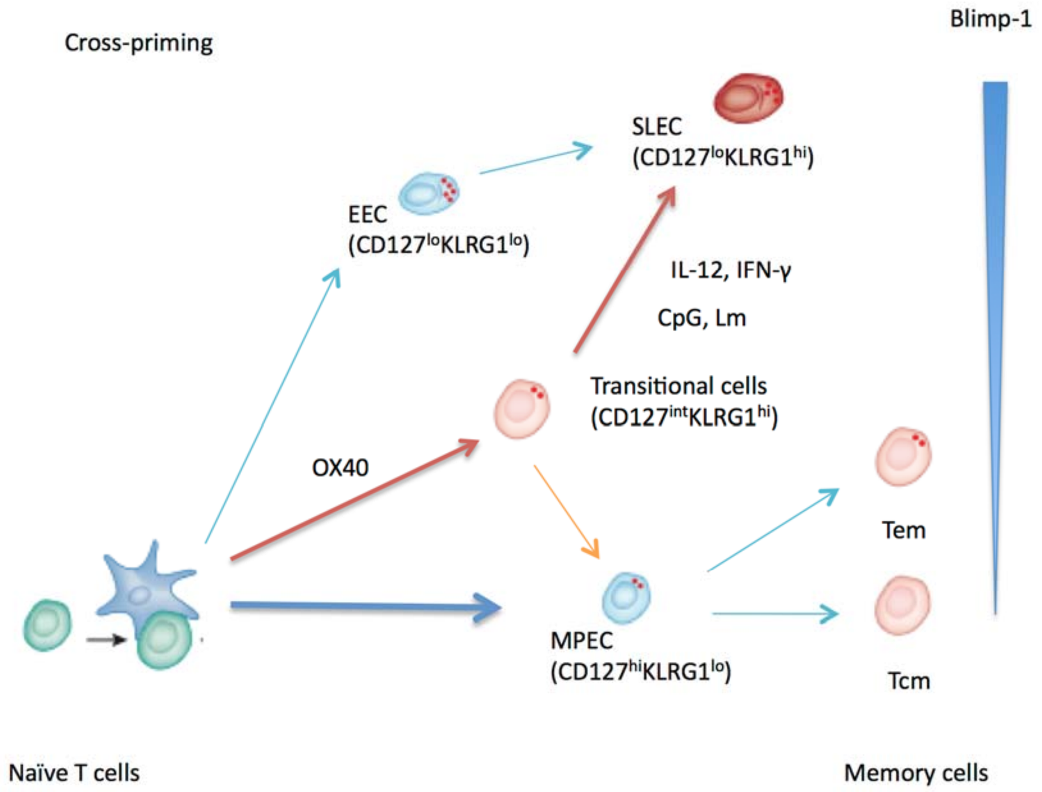

Supplementary Figure 2

A

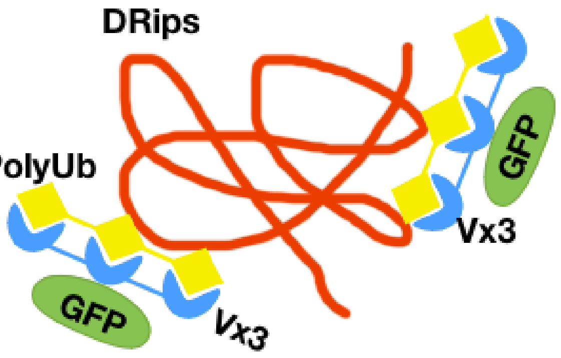

B

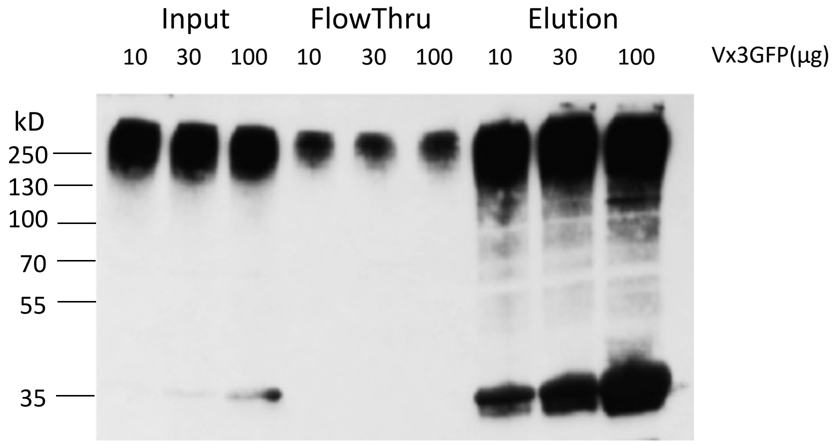

Supplementary Figure 3

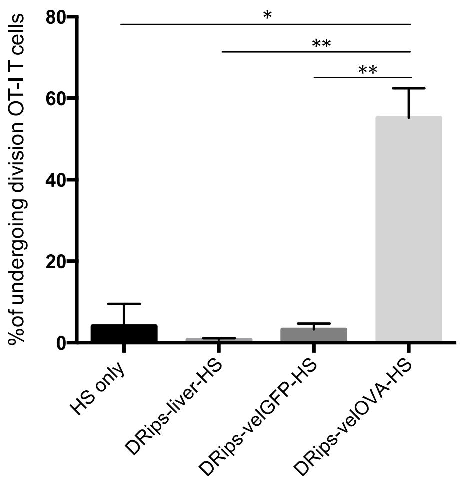

Supplementary Figure 4

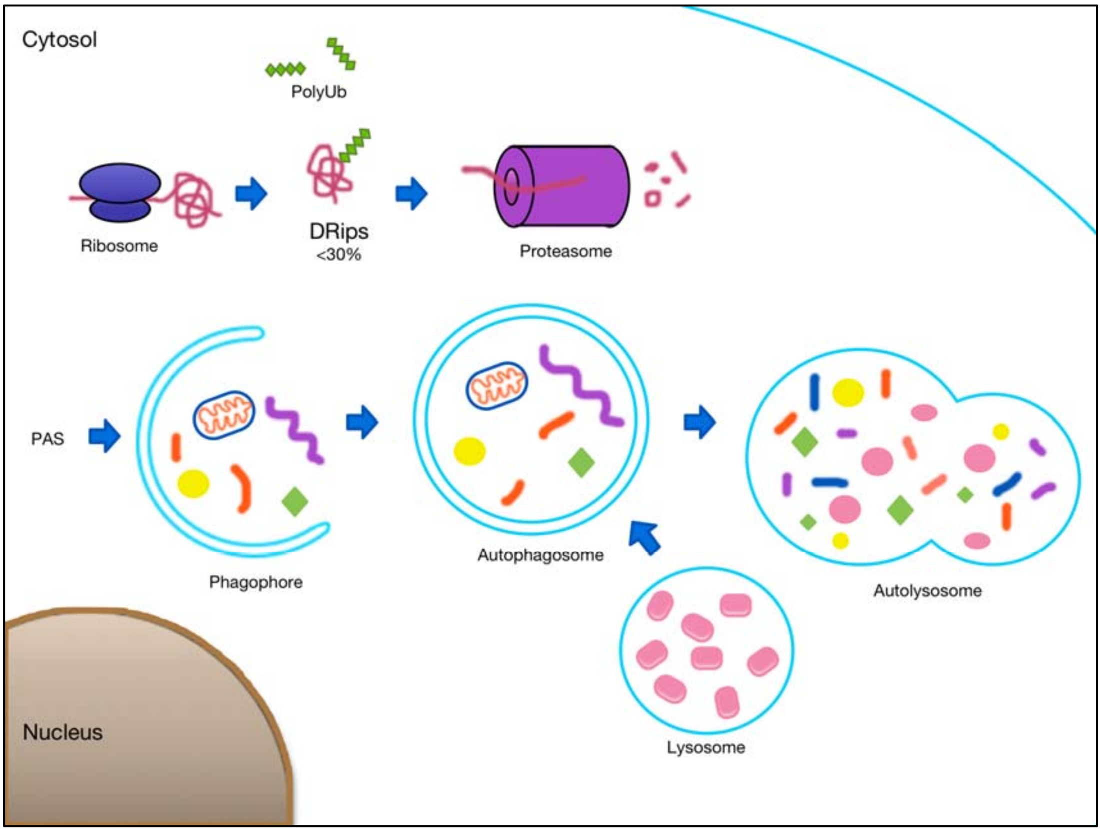

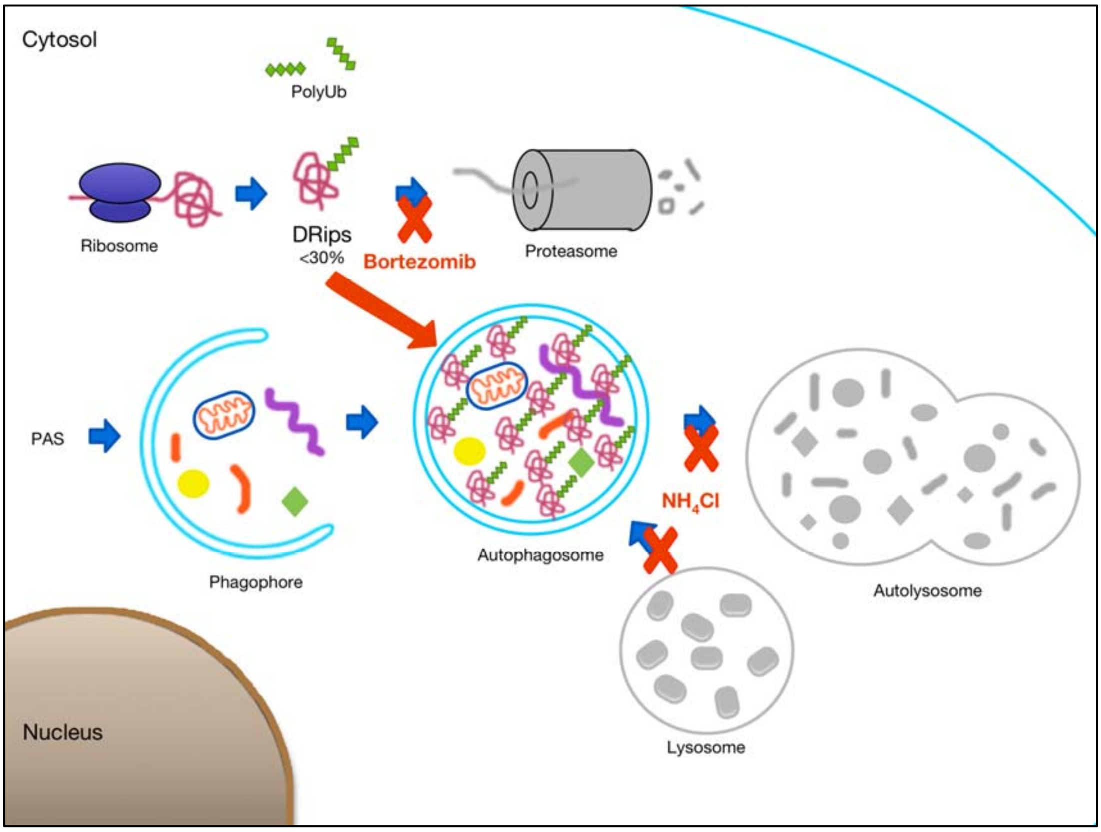

Supplement: Supplementary Information [file srep37558-s1.pdf]
